# Identification and genetic engineering of pneumococcal capsule-like polysaccharides in commensal oral streptococci

**DOI:** 10.1128/spectrum.01885-23

**Published:** 2024-03-15

**Authors:** Ren Wu, Moon Nahm, Jinghua Yang, C. Allen Bush, Hui Wu

**Affiliations:** 1 Department of Pediatric Dentistry, University of Alabama at Birmingham, School of Dentistry, Birmingham, Alabama, USA; 2 Department of Medicine, University of Alabama at Birmingham, School of Medicine, Birmingham, Alabama, USA; 3 Institute of Microbiology, Chinese Academy of Sciences, Beijing, China; 4 University of Chinese Academy of Sciences, Beijing, China; 5 Department of Chemistry and Biochemistry, University of Maryland at Baltimore, Baltimore, Maryland, USA; 6 Division of Biomaterial and Biomedical Sciences, Oregon Health & Science University School of Dentistry, Portland, Oregon, USA; Emory University School of Medicine, Atlanta, Georgia, USA

**Keywords:** *Streptococcus parasanguinis*, capsular polysaccharides, pneumococcal capsule, serotype switching, genetic engineering

## Abstract

Capsular polysaccharides (CPS) in *Streptococcus pneumoniae* are pivotal for bacterial virulence and present extensive diversity. While oral streptococci show pronounced antigenicity toward pneumococcal capsule-specific sera, insights into evolution of capsule diversity remain limited. This study reports a pneumococcal CPS-like genetic locus in *Streptococcus parasanguinis*, a predominant oral *Streptococcus*. The discovered locus comprises 15 genes, mirroring high similarity to those from the Wzy-dependent CPS locus of *S. pneumoniae*. Notably, *S. parasanguinis* elicited a reaction with pneumococcal 19B antiserum. Through nuclear magnetic resonance analysis, we ascertained that its CPS structure matches the chemical composition of the pneumococcal 19B capsule. By introducing the glucosyltransferase gene *cps19cS* from a pneumococcal serotype 19C, we successfully transformed *S. parasanguinis* antigenicity from 19B to 19C. Furthermore, substituting serotype-specific genes, *cpsI* and *cpsJ*, with their counterparts from pneumococcal serotype 19A and 19F enabled *S. parasanguinis* to generate 19A- and 19F-specific CPS, respectively. These findings underscore that *S. parasanguinis* harbors a versatile 19B-like CPS adaptable to other serotypes. Remarkably, after deleting the locus’s initial gene, *cpsE*, responsible for sugar transfer, we noted halted CPS production, elongated bacterial chains, and diminished biofilm formation. A similar phenotype emerged with the removal of the distinct gene *cpsZ*, which encodes a putative autolysin. These data highlight the importance of *S. parasanguinis* CPS for biofilm formation and propose a potential shared ancestry of its CPS locus with *S. pneumoniae*.

**IMPORTANCE:**

Diverse capsules from *Streptococcus pneumoniae* are vital for bacterial virulence and pathogenesis. Oral streptococci show strong responses to a wide range of pneumococcal capsule-specific sera. Yet, the evolution of this capsule diversity in relation to microbe-host interactions remains underexplored. Our research delves into the connection between commensal oral streptococcal and pneumococcal capsules, highlighting the potential for gene transfer and evolution of various capsule types. Understanding the genetic and evolutionary factors that drive capsule diversity in *S. pneumoniae* and its related oral species is essential for the development of effective pneumococcal vaccines. The present findings provide fresh perspectives on the cross-reactivity between commensal streptococci and *S. pneumoniae*, its influence on bacteria-host interactions, and the development of new strategies to manage and prevent pneumococcal illnesses by targeting and modulating commensal streptococci.

## INTRODUCTION

Capsular polysaccharides (CPS) produced by invasive *S. pneumoniae* are pivotal virulence factors in human infections. The effectiveness of capsule vaccinations against pneumococcal diseases, including pneumonia, underscores the significance of these capsules (1). Predominantly, unencapsulated strains of *S. pneumoniae* are avirulent. Historically, the presence of pneumococcal capsules was thought to differentiate *S. pneumoniae* from its closely related commensal oral streptococci counterparts. With over 91 structurally unique CPS identified in *S. pneumoniae* (2), understanding this diversity is imperative for the progressive design and development of pneumococcal vaccines. Evolving CPS serogroups, especially under the influence of vaccinations, highlight the dynamic genetic interexchange abilities within pneumococci (3–5). The varied capsules in pneumococci determine the bacteria’s interactions with its host (6). However, the intricacies of CPS evolution and capsule morphogenesis in pneumococci remain enigmatic.

Due to the advances in the next-generation sequencing technologies, a large number of bacterial genomes, including *S. pneumoniae* and commensal oral streptococci, have been sequenced and made publicly accessible. Studying CPS biosynthetic pathways among these genomes magnifies the diverse nature of CPS loci. Scrutiny of over 90 CPS loci from pneumococci juxtaposed with loci from 90 other oral streptococcal strains reveals shared CPS synthetic pathways with significant diversities. In *S. pneumoniae*, two distinct CPS synthesis pathways are documented: the prevalent Wzy-dependent pathway and the rarer synthase-dependent pathway (confined to two serotypes, type 3 and type 37). The Wzy-dependent loci are distinguished by two characteristic proteins: Wzy, an oligosaccharide repeat unit polymerase (CpsI), and Wzx, an oligosaccharide transporter(CpsJ) (7). The expansive CPS operon consists of four invariant regulatory proteins succeeded by a plethora of variable glycosyltransferases. Insightful studies on CPS loci from other pathogenic streptococci like *Streptococcus agalactiae* (8, 9), *Streptococcus suis* (10), and *Streptococcus iniae* (11) have revealed their crucial role in bacterial virulence. It is intriguing to note that the Wzy-dependent pathway also manifests in commensal oral streptococci, such as *Streptococcus mitis*, *Streptococcus oralis* (12), *Streptococcus gordonii* (13, 14), and others. A myriad of these commensal streptococci project antigenic mimicry with certain pneumococcal capsules, like serotypes 2, 5, 16A, 18F, 19C, 33A, 33D, 36, and 45 (15). This raises pertinent queries regarding the evolutionary trajectory of CPS via gene exchanges within *S. pneumoniae* strains and between *S. pneumoniae* and its commensal streptococci. Predominant theories suggest a unidirectional gene transfer from *S. mitis* to *S. pneumoniae* (16). Acquiring genes from oral streptococci could potentially diversify gene reservoirs, thereby catalyzing the remarkable capsular diversity seen in *S. pneumoniae*. Additionally, the alterations of the capsule types distribution in pneumococcal strain post-vaccination (17, 18) may inadvertently influence some commensal streptococci that resonate antigenically with *S. pneumoniae* capsules. However, the possibility and implications of such genetic exchanges, considering *S. pneumoniae*’s adeptness at genetic interchange, remain speculative. Whether this recognized antigenic overlap influences other facets of bacteria-host interactions mandates comprehensive study.

*Streptococcus parasanguinis* is an early colonizer of the oral cavity, which significantly contributes to the oral biofilm ecosystem (19). It now stands as the predominant oral *Streptococcus* (20) and has been associated with the etiology of infective endocarditis in humans (21, 22) and animals (23, 24). Analogous to *S. gordonii* and *S. oralis*, *S. parasanguinis* is typically unencapsulated. Up to now, the exploration of the CPS locus and structure from *S. parasanguinis*, particularly in relation to pneumococcal capsules, remains uncharted. This study reports the discovery of a CPS-like locus in *S. parasanguinis* FW213. We discern that this locus orchestrates the synthesis of a CPS identical to a 19B pneumococcal capsule. Moreover, it harbors a novel gene encoding a putative autolysin. Via mutagenesis and complements, we determined that the locus is indispensable for biofilm formation and elucidated the effects of *cps* genes on cell division and bacterial chain dimensions. Importantly, the *S. parasanguinis* capsule, reminiscent of its pneumococcal counterpart, exhibits flexibility, enabling genetic engineering to mimic diverse pneumococcal subtypes. This revelation implies a potential evolutionary trajectory where oral streptococci could influence the swift capsule evolution seen in *S. pneumoniae*, a characteristic hitherto overlooked.

## RESULTS

### Identification and comparison of CPS loci

**Fig 1:**
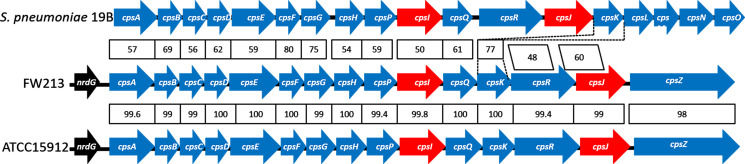
Comparison of the CPS loci from *S. parasanguinis and S. pneumoniae* 19B. The percentage of amino acid sequence identity of CPS homologs between the FW213 locus and pneumococcal 19b is shown. Dotted lines highlight the *cpsK* homolog from FW213 and 19B. The polymerase *cpsI* (*wzy*) and flippase *cpsJ* (*wzx*) are highlighted in red.

A BLAST search of *S. parasanguinis* FW213 genome unveiled a capsular polysaccharide biosynthetic gene cluster (*cps*) consisting of 15 open reading frames (ORFs). The first five genes (*cpsA*, *cpsB*, *cpsC*, *cpsD*, and *cpsE*) and the last gene (*cpsZ*) share a high degree of similarity to *cps* genes from other oral streptococci. Conversely, the remaining nine genes resemble their homologs from *S. pneumoniae* more closely. Strikingly, the gene order mirrors that of the *S. pneumoniae* type 19B CPS cluster, with the only exception being the location of *cpsK* (Fig. 1). Three genes, *cpsP*, *cpsQ*, and *cpsR*, are serotype specific and are exclusive to types 19B and 19C of *S. pneumoniae* (Fig. 1) but are absent in 19A and F (Fig. S4). Based on gene product alignment, we reason that FW213 harbors a Wzy-dependent CPS cluster. The four genes (*cpsL*, *cpsM*, *cpsN*, and *cpsO*) located in the 3′ region are crucial for the synthesis of the dTDP-Rha precursor for CPS production (25). Such structural organization is conserved among rhamnose-containing CPS clusters of *S. pneumoniae* types 1, 2, 6B, 19, and 23F (26). However, the dTDP-Rha synthesis genes (*rml*) are not present within this locus in FW213. Indeed, glucose-1-phosphate thymidylyltransferase (*rmlA*), dTDP-4-keto-6-deoxyglucose-3,5-epimerase (*rmlC*), and dTDP-glucose-4,6-dehydratase (*rmlB*) are situated in a separate locus on the FW213 chromosome. Meanwhile, a singular dTDP-4-dehydrorhamnose reductase (*rmlD*) is part of another gene cluster. The concluding gene, designed as *cpsZ*, encodes an N-acetylmuramidase-like (AtlA-like) protein. Found in lactic acid bacteria, this family of proteins is postulated to be a putative autolysin, responsible for bacterial cell wall degradation. Although this protein remains uncharacterized, its coding gene frequently appears at the 3′ end of receptor polysaccharides clusters similar to those of *S. gordonii* (14).

An analysis of the fully annotated *S. parasanguinis* genomes available on NCBI showed that FW213 and ATCC15912 genomes exhibit a very high identity. A striking identity of over 99% emerges when aligning the CPS locus DNA sequences between FW213 and ATCC15912 (Fig. 1). Of 137 *S*. *parasanguinis* genome assemblies on NCBI, only the strains ATCC15912 and FW213 are completed genomes. When the cpsA nucleotide sequence was blasted against the *S. parasanguinis* whole-genome shotgun contigs available, 135 out of 137 *S*. *parasanguinis* genome assemblies were found to have this conserved gene. This underscores the prevalent presence of CPS loci in numerous *S. parasanguinis* strains. Moreover, 17 genome assemblies contain the 19B serotype-specific gene *cspR*, highlighting the occurrence of this unique serotype CPS-like polysaccharides in other *S. parasanguinis* strains. Although AtlA-like genes are not universally conserved at the nucleotide sequence level, multiple AtlA-like genes have been identified in *S. parasanguinis*. Many *S. parasanguinis* genomic assemblies yielded significant matches when blasted with the AtlA-like gene sequence. However, more in-depth research is required to ascertain its locations and functions, especially in relation to the CPS loci. Notably, several other *S. parasanguinis* assembly genomes, including ATCC903 and F0405, feature a distinct putative CPS cluster followed by an AtlA-like gene.

### The antigenicity of FW213 CPS

**Fig 2:**
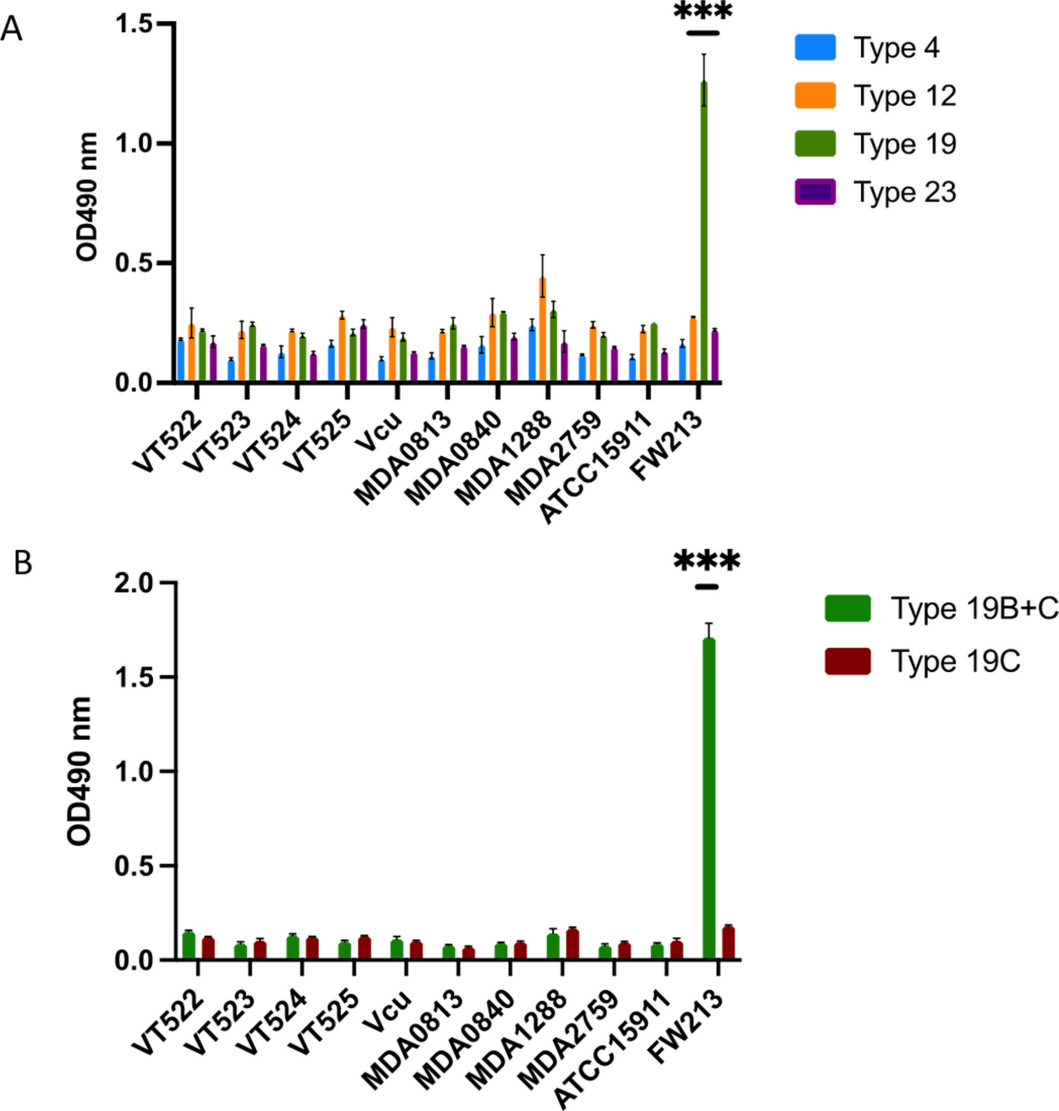
Antigenicity of diverse *S. parasanguinis* strains determined by antisera for select pneumococcal capsules. BactELISAs were performed using antisera against *S. pneumoniae* serotype 4, 12, 19, and 23 (**A**) and *S. pneumoniae* serotype 19B and C (**B**) capsules. Samples were analyzed in triplicate, with their reactivity displayed as OD values of 490 nm. Error bars indicate standard deviations.

Given that nine serotype-specific CPS genes from *S. parasanguinis* closely resemble *S. pneumoniae* cps genes, antisera against S. *pneumoniae* types 4, 12, 19, and 23 were used to evaluate sera reactivities of *S. parasanguinis* FW213, ATCC15911, and nine clinical isolates (VT522, VT523, VT524, VT525, Vcu, MDA0813, MDA0840, MDA1288, and MDA2759). Only FW213 showed a strong reaction with the serotype 19 antiserum (Fig. 2A). To pinpoint antigenic specificity, eight different streptococci known to express cell surface polysaccharides were tested using the serotype 19 antiserum. Again, only FW213 demonstrated the presence of a specific type 19-like CPS (Fig. S5). Additionally, antiserum 19A, 19F (Fig. S5), 19B&C, and 19C were employed to discern the sub-serotypes. FW213 showed a robust reaction only with 19B&C and not with 19C (Fig. 2B), implying that *S. parasanguinis* FW213 produces a CPS similar to type 19B.

### Determination of structure of FW213 CPS by NMR

To characterize the structure of CPS expressed by FW213, we purified CPS and subjected it to high-resolution nuclear magnetic resonance (NMR) analysis, comparing it to the pneumococcal 19B capsule. The NMR data confirmed all the linkages for pneumococcal 19B Cps (Statens Serum Institut, Copenhagen) to match the structure proposed earlier (27). The data (Table 1) revealed subtle differences between the chemical shift values reported previously and our findings. However, more importantly, our data for *S. pneumonia*e CPS 19B closely align with the data for the *S. parasanguinis* FW213 cell wall polysaccharide. The heteronuclear multiple bond correlation (HMBC) data also confirm that the glycosidic linkage positions for both polysaccharides are consistent. The phospho-diester linkage between A1 and E4 was determined by ^1^H-^31^P heteronuclear single quantum coherence (HSQC). Hence, we conclude that both polysaccharide structures are identical.

**TABLE 1:** *S. parasanguinis*/*S. pneumoniae* 19B NMR data

Strains	Residues	Chemical shifts (ppm)					
		1-H 1-C	2-H 2-C	3-H 3-C	4-H 4-C	5-H 5-C	6-H 6-C
*S. parasanguinis* FW213	α-Rha (A)	5.372 97.014	3.995 71.592	3.921 70.393	3.695 80.268	3.905 69.153	1.318 17.808
*S. pneumoniae* 19B (27)		5.370 97.30	3.990 72.30	3.940 71.50	3.700 80.60	3.910 69.40	1.320 18.20
*S. pneumoniae* 19B^*a*^		5.372 97.00	3.996 71.60	3.943 70.39	3.698 80.26	3.913 69.15	1.322 17.81
*S. parasanguinis* FW213	β-Rib (B)	5.290 109.08	4.105 75.89	4.123 71.82	4.023 83.32	3.685, 3.822 63.89	
*S. pneumoniae* 19B (27)		5.290 109.40	4.100 76.20	4.120 72.30	4.020 83.70	3.690, 3.830 64.20	
*S. pneumoniae* 19B^*a*^		5.292 109.06	4.105 75.89	4.124 71.79	4.024 83.28	3.691, 3.823 63.88	
*S. parasanguinis* FW213	β-ManNAc (C)	5.012 100.40	4.713 50.44	3.979 74.71	3.909 71.57	3.522 76.49	3.916, 3.993 60.41
*S. pneumoniae* 19B (27)		5.030 100.80	4.710 50.80	3.970 75.10	3.900 71.90	3.510 76.10	3.69, 3.96 60.70
*S. pneumoniae* 19B^*a*^		5.018 100.39	4.717 50.43	3.993 74.73	3.909 71.58	3.521 76.48	3.930, 4.008 60.41
*S. parasanguinis* FW213	α-Rha (D)	4.953 97.33	3.759 71.31	3.941 71.23	3.508 80.30	4.253 67.75	1.286 17.64
*S. pneumoniae* 19B (27)		4.940 97.70	3.760 71.50	3.920 71.60	3.510 80.60	4.240 68.10	1.290 17.90
*S. pneumoniae* 19B^*a*^		4.962 97.33	3.758 71.31	3.928 71.23	3.506 80.28	4.269 67.75	1.288 17.64
*S. parasanguinis* FW213	β-ManNAc (E)	4.840 100.16	4.573 53.82	3.985 71.96	4.076 72.97	3.560 76.49	3.831, 3.933 61.30
*S. pneumoniae* 19B (27)		4.830 100.50	4.570 54.10	3.980 71.90	4.070 73.30	3.560 76.80	3.830, 3.940 61.60
*S. pneumoniae* 19B^*a*^		4.847 100.17	4.575 53.82	3.994 71.95	4.075 72.96	3.565 76.48	3.833,3.935 61.31
*S. parasanguinis* FW213	β-Glc (F)	4.496 102.35	3.214 74.47	3.678 74.68	3.374 80.93	3.519 75.76	3.701, 3.899 62.09
*S. pneumoniae* 19B (27)		4.480 102.50	3.200 74.70	3.670 75.00	3.350 81.30	3.520 76.80	3.690, 3.890 62.50
*S. pneumoniae* 19B^*a*^		4.494 102.35	3.215 74.46	3.687 74.69	3.366 80.95	3.519 75.77	3.695, 3.911 62.08

^*a*^ *S. pneumoniae* 19B in this study.

The only distinction between the spectra of *S. parasanguinis* (Fig. S1) and *S. pneumoniae* 19B lies in the absence, in the former, of phosphocholine peaks at 3.23 and 54.87 ppm. This is coupled with the absence of peaks from a methylene group at 4.32 and 60.30 ppm, which are characteristic of the pneumococcal common antigen, C-polysaccharide. This particular polysaccharide is often isolated as a contaminant in most samples of *S. pneumoniae*, and its NMR properties have been well documented (28). The NMR structural studies unveil that FW213 produces a CPS indistinguishable from the pneumococcal 19B capsule (Fig. S3).

### Identification of *cps* genes as serotype determinants of the FW213 CPS

**Fig 3:**
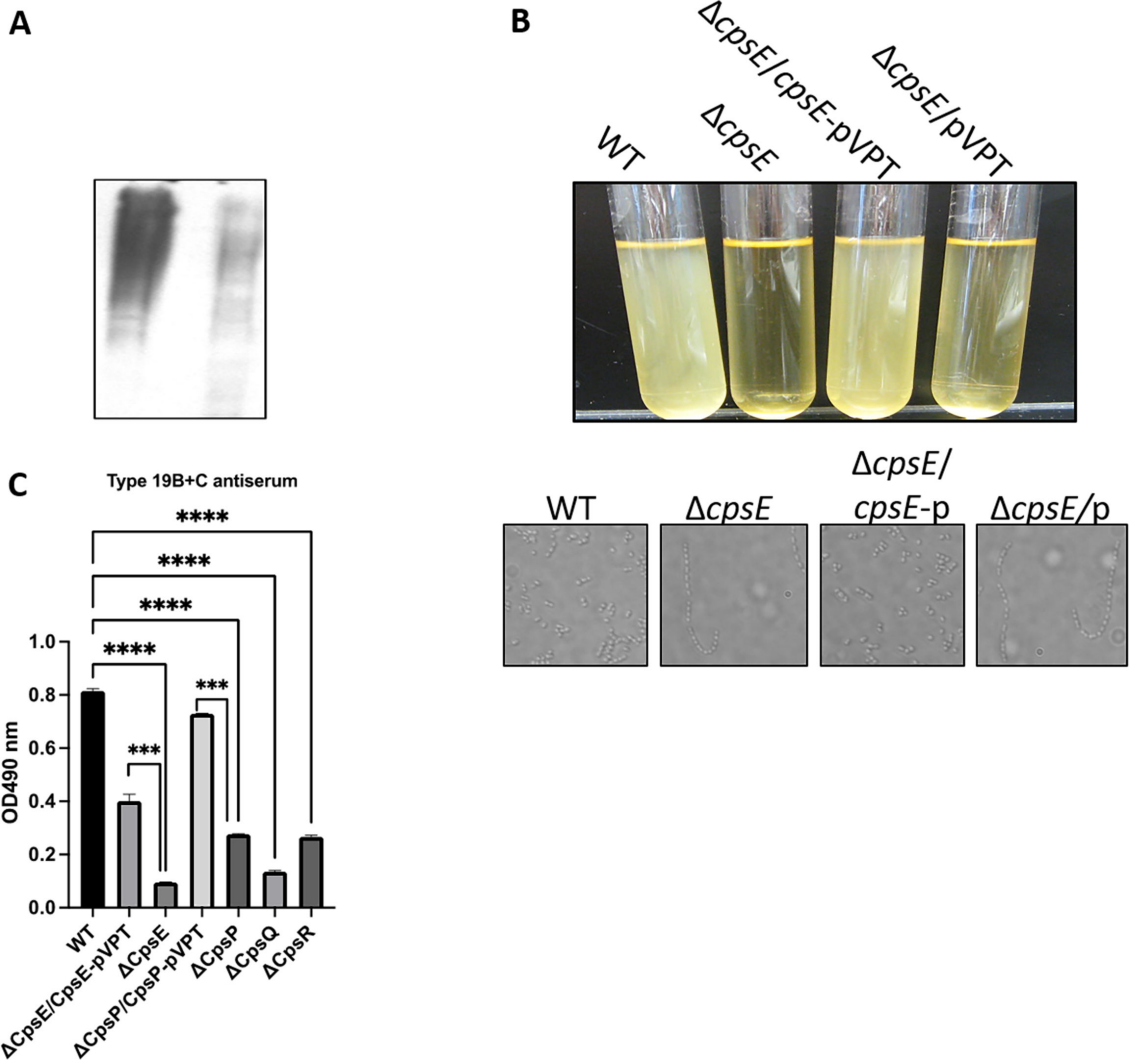
Characterization of *cps* mutants and their respective complements. (A) Western blotting analysis of CPS. FW213 and its derivatives were grown to OD_470 nm_ = 0.8. Cell lysates were then prepared and analyzed by Western blot using antiserum 19B+C. (B) Phenotypic characterization of *S. parasanguinis* FW213 and its variants. Bacteria were grown overnight in test tubes. (C) Morphology of FW213 and its derivatives. Bacteria grown to OD_470 nm_ = 1.0 were examined using phase-contrast microscope at 100× magnification. Representative micrographs are shown. (D) CPS levels of *S. parasanguinis* were determined by ELISA. BactELISAs were performed using antisera against *S. pneumoniae* type 19B+C. Error bars indicate standard deviations.

Given that FW213 reacts with 19B serotype antiserum, we hypothesized that the FW213 CPS cluster determines this reactivity. To investigate this, we first constructed a non-polar deletion in the gene encoding the initial glycosyltransferase CpsE. As expected, the ∆*cpsE* mutant no longer reacted with 19B antibody (Fig. 3A), indicating that the FW213 CPS cluster is functional and accountable for the production of the 19B-like serotype CPS. Upon further characterization of the ∆*cpsE* mutant, a marked morphological change was noted. Bacterial cells aggregated, precipitating at the bottom of the test tube during the late growth phase (Fig. 3B, upper panel). Predominantly, the mutant appeared in long chains (more than 10 cells in length) in contrast to diplococcal morphology of wild-type cells (Fig. 3B, bottom panel). These phenotypic changes, coupled with the deficient CPS, were rescued by genetic complementation (Fig. 3). This suggests that the cell surface CPS influences cell division and cell envelope biogenesis.

**Fig 4:**
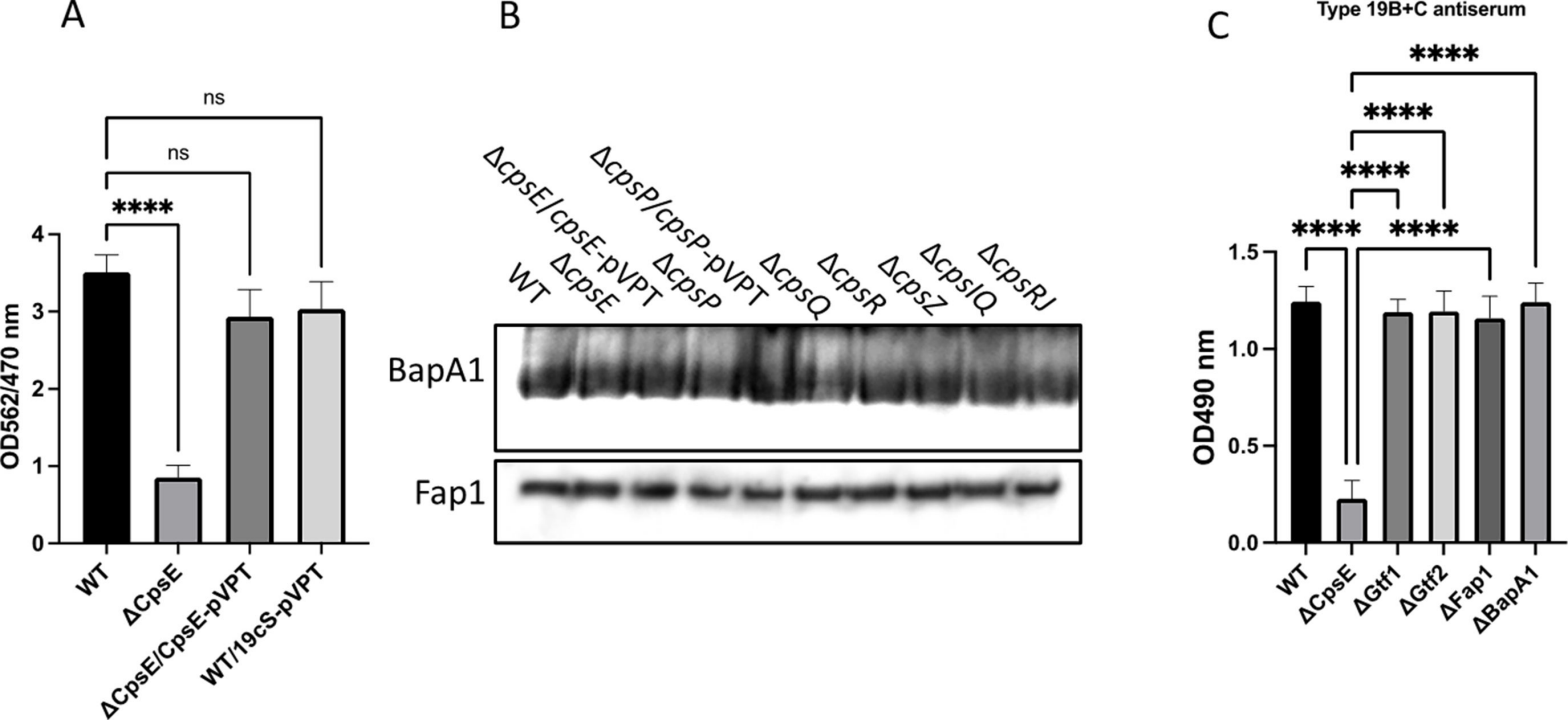
Biofilm formation by *S. parasanguinis.* (A) CPS deficiency affects *S. parasanguinis* biofilm formation. Biofilms were assayed in triplicate, and biofilm biomass was shown as normalized OD values at 562/470 nm. Error bars indicate standard deviations. (**B**) CPS mutants do not affect known biofilm determinants, Fap1 and BapA1. Western blot analysis was conducted using the mAbE42 antibody for Fap1 and a polyclonal antiserum against BapA1. (**C)** Cps levels are unaltered in *fap1* and *bapA1* mutants. BactELISA tests were conducted using antisera against *S. pneumoniae* types 19B+C and 19C. Signals measured at OD_490 nm_ represent the relative reactivity of *S. parasanguinis* variants with pneumococcal antisera. Samples were tested in triplicate, and reactivity is shown in OD values at 490 nm. Error bars indicate standard deviations.

To further investigate the contribution of the locus, we constructed mutants in three serotype-specific putative genes *cpsP* and *cpsQ* (both responsible for the transferring of side-chain sugars to the repeat units of CPS) and *cpsR* (which supports the NDP-ribose synthesis pathway). Production of 19B-like CPS was inhibited in all three mutants (Fig. 3C), as measured by enzyme-linked immunosorbent assay (ELISA), and they displayed a long-chain morphology (Fig. S2). Importantly, colony-forming unit counts indicated that these mutations did not affect the cell viability. Bacterial cultures from wild type and mutants, grown at an optical density (OD) of 0.8 at 470 nm, were harvested and sonicated for four 30-second intervals and then subjected to serial dilution before being plated on Todd Hewitt broth (THB) agar plates for colony counting. These findings suggest that overall bacterial growth remains unaffected in these mutants, confirming that cell viability and general bacterial growth were not altered by these mutations. A reduction in CPS has been previously linked to increased *S. pneumoniae* biofilm formation *in vitro* (29). Therefore, we investigate the role of CPS in FW213 biofilm using the *cpsE* mutant. The *cspE* mutant significantly reduced biofilm formation, but this reduction was readily reversed when complemented with its full-length gene (Fig. 4A). Taken together, these findings suggest that FW213 CPS plays a role in the cell envelope biogenesis and biofilm formation.

In *S. parasanguinis*, biofilm formation is known to be mediated by two cell wall-anchored proteins. Fap1 is a serine-rich repeat glycoprotein required for the biofilm formation both *in vitro* and *in vivo* (30), while BapA1 is another high-molecular-weight protein involved in the biofilm formation (31, 32). To determine whether the effect of CPS on the biofilm formation is modulated through either Fap1 or BapA1, we assessed the impact of CPS deficiency on Fap1 and BapA1. The production of both Fap1 and BapA1 was unaffected by the CPS mutants (Fig. 4B). Conversely, a deficiency in Fap1 or defects in Fap1 glycosylation (*gtf1* and *gtf2*) (33, 34) did not influence CPS levels (Fig. 4C). These findings indicate that the CPS-mediated biofilm formation operates independently of two characterized biofilm-related proteins.

### CPS serotype switching from 19B to 19C

**Fig 5:**
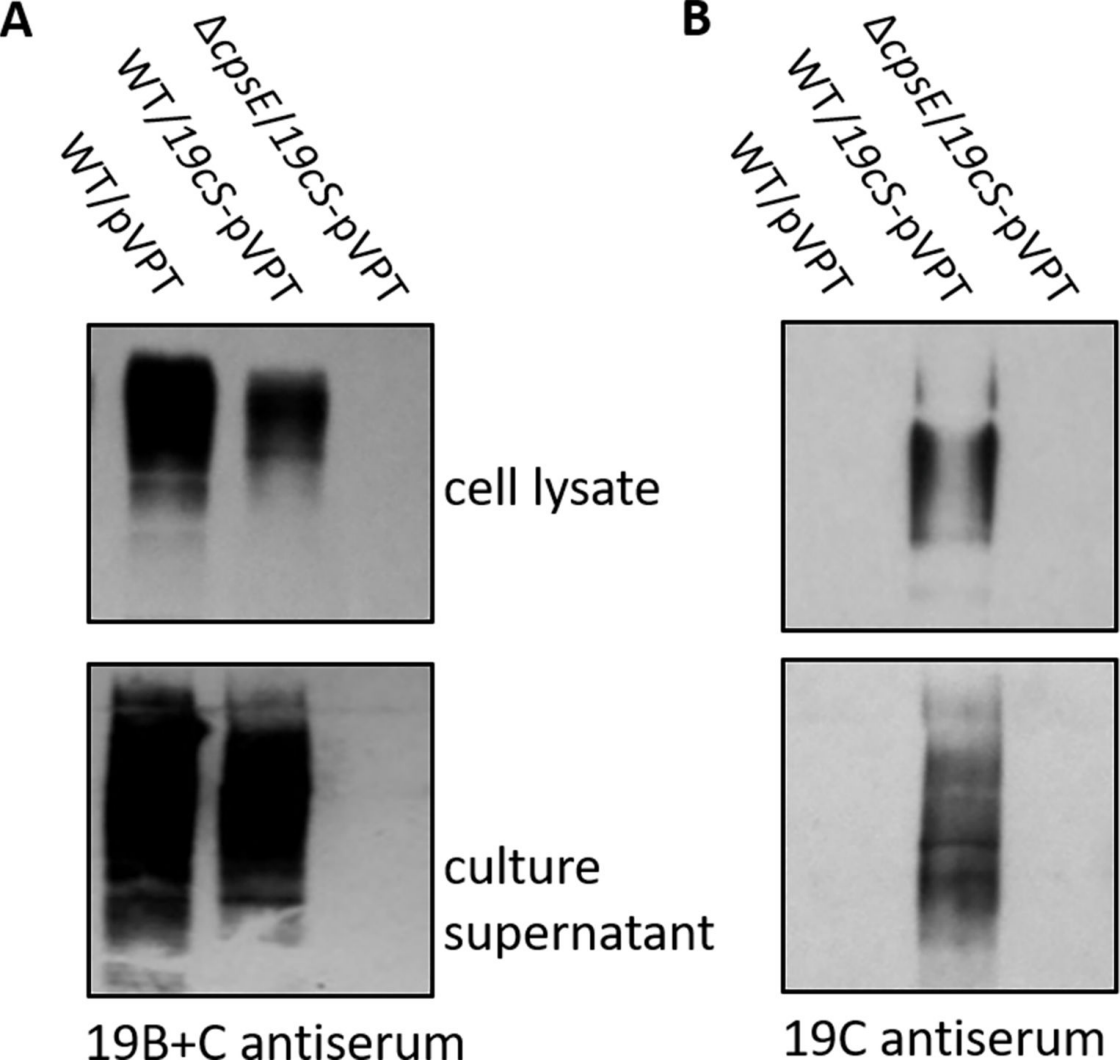
Switching CPS serotype from 19B to 19C in *S. parasanguinis*. Wild-type *S. parasanguinis* FW213 cells were transformed with cps19cS from *S. pneumoniae*. Cells were grown to OD_470 nm_ = 0.8. Cell lysates and culture supernatants were then prepared to examine CPS by Western blot using *S. pneumoniae* antiserum 19B+C (**A**) and 19C (**B**).

The distinction between the *S. pneumoniae* 19B and 19C capsules lies in an extra glucose found in the 19C pneumococcal capsule, which is transferred by a glucosyltransferase gene *cps19cS* (25). To assess whether we could convert FW213 19B-like CPS to 19C-like CPS, we cloned *cps19c* from *S. pneumoniae* and transferred it into FW213. Following this genetic modification, the CPS serotype successfully transitioned to 19C (Fig. 5A and B). However, overexpressing *cps*19cS in the ∆*cpsE* mutant did not yield CPS that reacted with either 19B or 19C (Fig. 5A and B). This alteration had no effect on bacterial biofilm formation (Fig. 4A). These data demonstrate that the serotype-specific gene, *cps19cS*, retains its functional conversion; the FW213 *cps* locus is akin to the pneumococcal *cps* locus.

### Switching CPS serotype in FW213 by engineering *cpsI* and *cpsJ*

**Fig 6:**
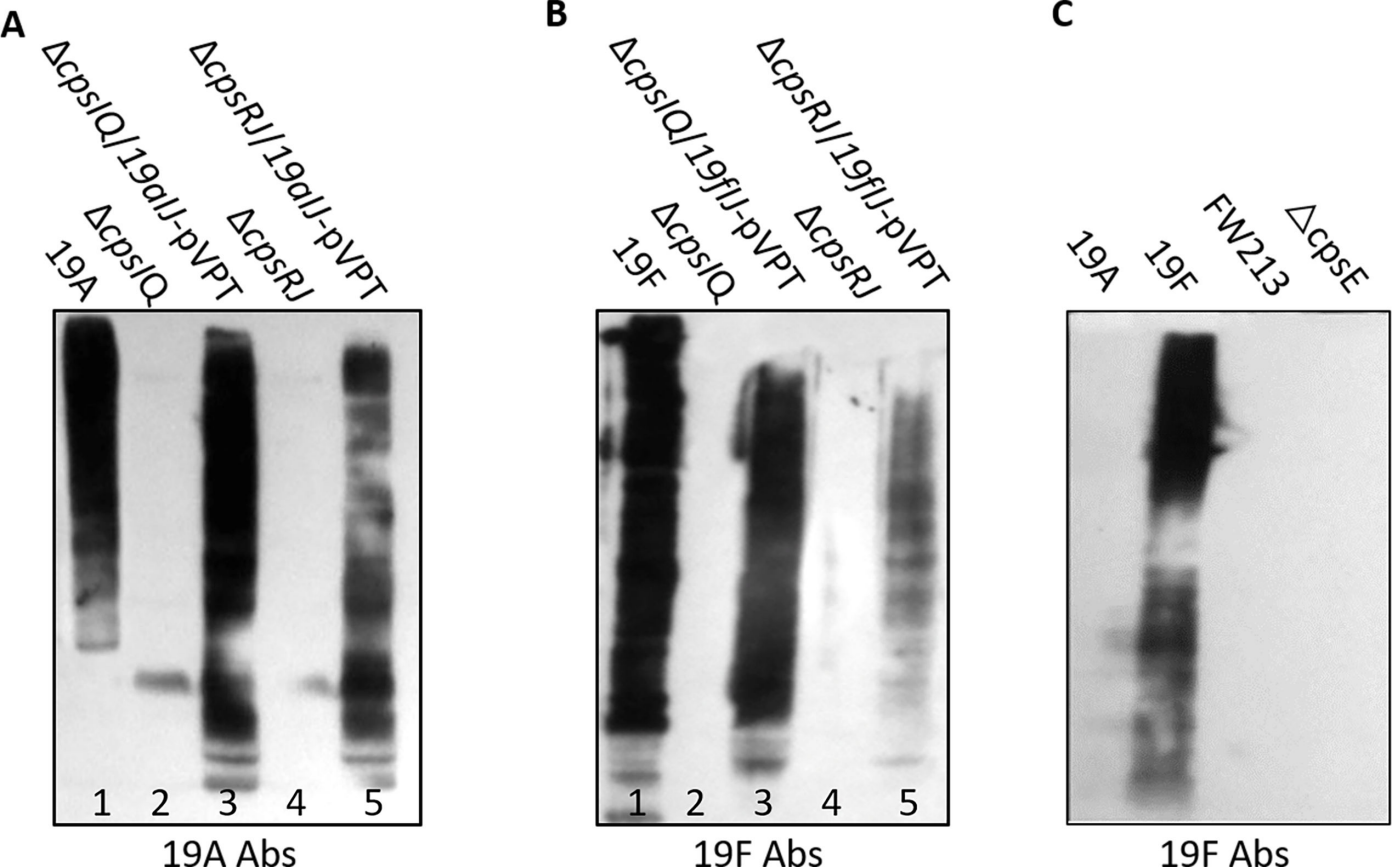
Switching CPS serotype from 19B to 19A and 19F in *S. parasanguinis*. Specific genes for select serotype were deleted and replaced with corresponding 19A, 19F-specific genes from *S. pneumoniae*. Engineered strains underwent Western blot analysis to determine their CPS serotypes. Cell lysates, prepared from cells grown to an OD_470 nm_ = 0.8 were separated by 8% SDS-PAGE and then subjected to Western blotting using *S. pneumoniae* 19A (**A**) and 19F (**B**) antiserum. *S. pneumoniae* 19A, 19F, *S. parasanguinis* FW213, and its cpsE mutant were probed with pneumococcal 19F antibody (**C**).

Pneumococcal CpsI functions as a polymerase, which directs the linkage difference between 19A and 19F (35). CpsJ is a putative flippase, determining the transfer of either one or two ManNAc to the linear repeat unit of CPS (35), thus differentiating the pneumococcal 19A/F from 19B/C (Fig. 6C). The genes *cpsR* and *cpsQ* encode enzymes responsible for transferring side-chain sugars to the repeat unit (35), and these are unique to pneumococcal 19B/C. To engineer CPS biosynthesis in *S. parasanguinis*, we established two double mutants: ∆*cpsIQ* and ∆*cpsRJ*. These double mutants are anticipated to hinder the production of the original 19B-like CPS but should maintain the synthesis of the linear backbone (either Rha-ManNAc-Glc or ManNAc-Rha-ManNAc-Glc). By introducing two genes *cps19aIJ* from *S. pneumoniae*, we successfully converted FW213 CPS serotype to 19A using either ∆*cpsIQ* or ∆*cpsRJ* mutant as a recipient (Fig. 6A). Within whole cell lysates, introducing *cps19aIJ*-pVPT in the ∆*cpsIQ* mutant yield more 19A CPS than in the background of the ∆*cpsRJ* mutant (Fig. 6A, lane 3 versus lane 5). A similar outcome was observed when converting to the 19F serotype using *S. pneumoniae cps19fIJ*-pVPT in these two mutants (Fig. 6B, lanes 3 and 5). These results indicate that CpsI and CpsJ are interchangeable and capable of switching type 19 CPS. However, when combined, they are optimal for transporting and polymerizing the trisaccharide (Rha-ManNAc-Glc) in the FW213 double mutants.

### Autolysin-like CpsZ involved in cell division, CPS production, and biofilm formation

**Fig 7:**
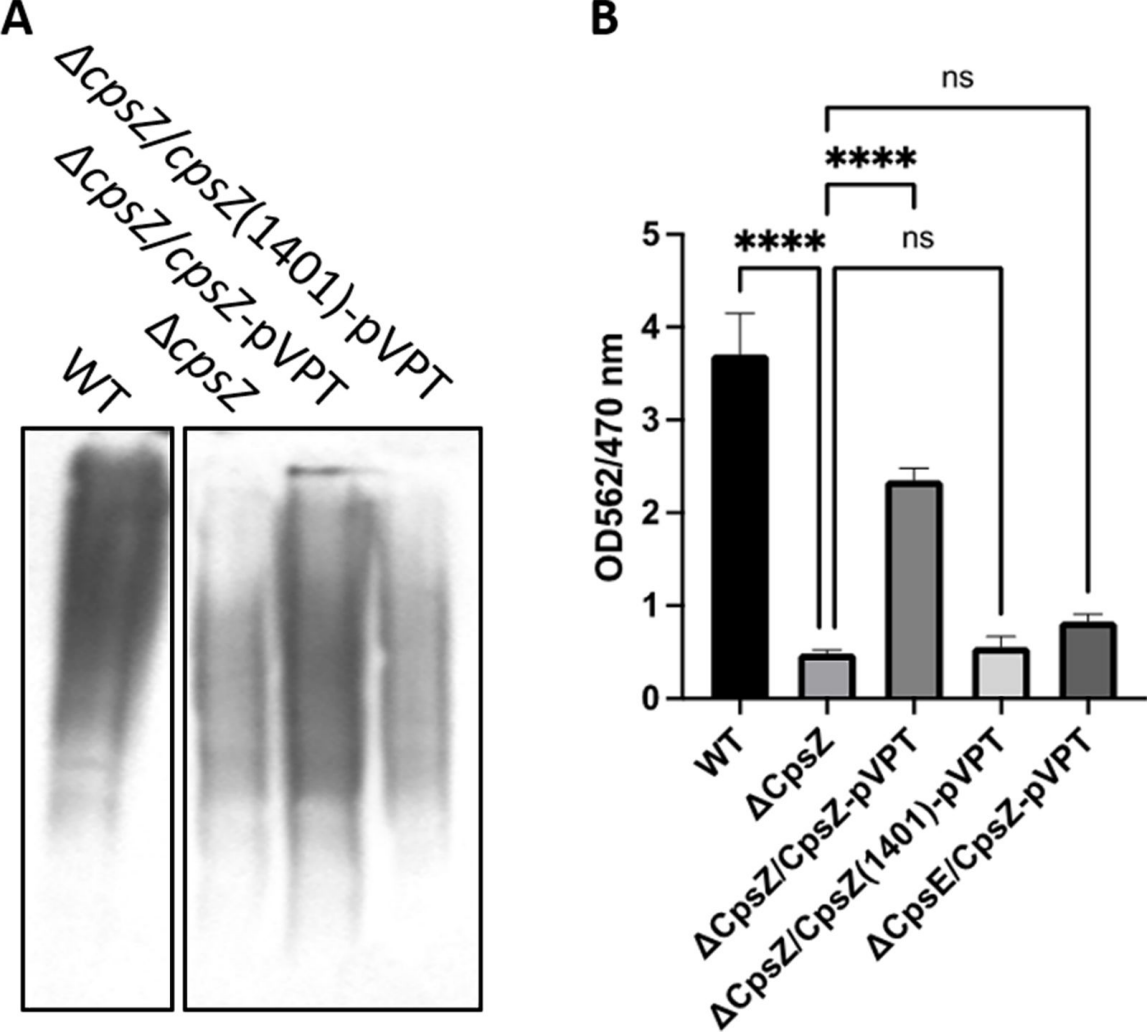
Characterization of the *cpsZ* mutant. Cell lysates from wild-type *S. parasanguinis* (WT), the *cpsZ* mutant, and its complement were used to evaluate CPS production (**A**) by Western blotting using *S. pneumoniae* antiserum and biofilm formation (**B**) analyzed using the crystal violet staining method.

Unlike the *S. pneumoniae* CPS locus, an *atlA-like cpsZ* gene is present in the FW213 *cps* locus. We initially assessed the role of CpsZ in production of CPS using Western blotting analysis on its mutant. The CPS level was markedly decreased in the CpsZ mutant, especially the high-molecular-weight CPS (Fig. 7A). Complementation restored the CPS level. These results suggest that CpsZ plays a crucial role in CPS polymerization. CpsZ shares homology with N-acetylmuramidase and is considered a putative autolysin. As expected, the ∆*cpsZ* mutant exhibited long chains (Fig. S2), suggesting its potential involvement in cell division and the cell envelope biogenesis. Given that CpsZ shares characteristics with cell wall hydrolases and is situated downstream of the CPS cluster, we postulated that CpsZ might account for the observed elongated chain phenotype of other mutants, including ∆*cpsE*, ∆*cpsP*, ∆*cpsR*, and ∆*cpsQ*. However, mutations in these *cps* genes did not influence the expression of *cpsZ* (Fig. S6), and complementation effectively reinstated wild-type phenotypes for all the mutants. This suggests that the mutations in cps genes did not exhibit polar effects on *cpsZ*, and the observed long-chain phenotype is directly linked to CPS production. To further determine how CpsZ might influence bacterial chain lengths, we overexpressed *cpsZ*-pVPT in these *cps* mutants and then monitored bacterial chain lengths. Overexpressing *cpsZ*-pVPT in these *cps* mutants noticeably shortened their chain lengths (Fig. S2). Additionally, the ∆*cpsZ* mutant reduced FW213 biofilm formation (Fig. 7B). CpsZ homologs from oral streptococci exhibit similarities in conserved domains, including the GBS_Bsp_like domain. The repeats of Bsp_like domain vary among oral streptococci (15). To investigate the role of the length of repeat units in *cpsZ*, we constructed a truncated *cpsZ* (1–1,401 bp) that contains only one GBS Bsp_like domain. This truncated CpsZ (1041) failed to rescue the ∆*cpsZ* mutant. However, the intact *cpsZ*-pVPT did enhance CPS production (Fig. 7A). This evidence suggests that a sufficient number of Bsp_like repeats are essential for maintaining its activity in both CPS biosynthesis and cell division.

## DISCUSSION

In this study, we discovered a 19B-like CPS produced by the oral streptococcus, *S. parasanguinis* FW213. While the 19B-like CPS is linked with cell wall and is also excreted into culture medium, it does not develop a dense capsule layer, akin to what is observed in *S. pneumoniae*. This is despite its structural and antigenic similarities with the pneumococcal 19B capsule. The FW213 CPS locus exhibits significant homology with the pneumococcal 19B *cps* locus. By deleting serotype-specific genes, *cpsE*, *cpsP*, *cpsQ*, and *cpsR*, we confirm their roles in determining the CPS serotype in *S. parasanguinis*. However, other disparities are evident. The *cps* genetic locus in various sequenced *S. parasanguinis* strains and in other oral streptococci is situated between two consistent genes, *nrdG* and *cpsZ*. In contrast, the dTDP-Rha biosynthetic *rml* genes, present in the pneumococcal *cps* locus, are positioned differently in *S. parasanguinis*—not adjacent to the *cps* locus in *S. parasanguinis*. The overall arrangement of the four *rml* genes is consistent among other oral streptococci that have similar CPS clusters (14, 36). It is yet to be determined if these differences influence the presentation of CPS on the cell surface. Nonetheless, when the FW213 19B CPS was switched to other pertinent subtypes, 19C/A/F, it exhibited strong similarity to pneumococcal capsules. The transition of 19B-like CPS of *S. parasanguinis* to 19C-like CPS was fully achieved by expressing the pneumococcal glucosyltransferase gene, *cps19c*S. In a similar fashion, the pneumococcal 19B can be effectively converted into 19C capsule (37). NMR analyses indicate that the CPS of FW213 possesses chemical structures identical to the *S. pneumoniae* 19B capsule. Moreover, we successfully switched the FW213 19B CPS to two other subtypes, 19A and F, by replacing two genes, either *cpsQ* or *cpsR*, responsible for adding side chains for 19B/C—these genes are absent in *S. pneumoniae* 19A/19F cluster. We replaced them with two specific genes, *cpsI* and *cpsJ*, which are crucial determinants for the 19A/F serotypes. The CPS loci for *S. pneumoniae* 19A and 19F are highly similar, both featuring the same trisaccharide (Rha-ManNAc-Glc) without the side chain. However, there is a unique linkage between repeat units (25), which is regulated by CpsI. The 19B and 19C capsules contain a tetrasaccharide backbone decorated with a bisaccharide side chain (25). By deleting either *cpsR* or *cpsQ*, this side chain is removed, paving the way for the transition to 19A/F. The successful alternation of FW213 using *S. pneumoniae cps* gene homologs underscores the close relationship of FW213 CPS with *S. pneumoniae* capsules. Within *S. pneumoniae*, serotype switches have been documented (37), as well as between *S. pneumoniae* and *S. gordonii* (38). Similarly, serotype switching via the substitution of a *cpsI* homolog has been observed in *S. agalactiae* (9). These studies validate the engineered transition achieved by swapping *cpsI* and /or *cpsJ* for their *S. pneumoniae* equivalents, reinforcing that both CpsI and CpsJ are functionally conserved and are pivotal in determining CPS.

Pneumococcal strains both in laboratory settings and in the wild are highly transformable and frequently exchanging their CPS loci (3, 39). The varied similarities and differences in CPS among different streptococci at the molecular and structural levels offer a lens through which to study evolution of CPS. While there are some studies highlighting antigenic similarities in surface polysaccharides between oral streptococci and *S. pneumoniae* (12, 15, 38, 40), our understanding of how these surface polysaccharides between oral streptococci and *S. pneumoniae* evolved remains imcomplete. There is speculation that *S. mitis* and *S. oralis* descended from an ancestral *S. pneumoniae* (12, 41). In our study, the resemblance of the *cps* genetic locus between *S. pneumoniae* 19B and *S. parasanguinis* FW213 resulted in the production of CPS with identical antigenic properties. Unlike *S. pneumoniae* and *S. gordonii*, *S. parasanguinis* FW213 is not naturally transformable. This raises the possibility that the cps cluster in *S. parasanguinis* is acquired from *S. pneumoniae* clusters, under host immune pressure or other environmental challenges. Given the intricate oral biofilm community’s capacity for cell-cell communication, coaggregation, and even genetic exchange, it offers a robust genetic environment that could facilitate gene transfer and the subsequent swapping and transition of CPS. An alternate theory posits that the CPS locus existed in a shared ancestor and was bequeathed to the contemporary *S. parasanguinis* and *S. pneumoniae* as they branched off evolutionarily into their current roles as commensal and pathogen, respectively.

CPS is crucial for biofilm formation in *S. parasanguinis*. However, in stark contrast, most *S. pneumoniae* CPS seems to impede biofilm formation (42). Upon bacterial invasion of host cells, there is a noticeable decrease in pneumococcal capsule production (43, 44). Interestingly, both low encapsulated *S. pneumoniae* and unencapsulated *Staphylococcus aureus* demonstrate enhanced biofilm persistence on host cells (43, 45). Broadly, while encapsulation amplifies bacterial invasive potential, it diminishes their adherence and biofilm-forming capabilities. Given that *S. parasanguinis* lacks encapsulation and possesses biofilm-forming capacity, it might be less invasive than the pathogenic *S. pneumoniae*. The potential role of *S. parasanguinis* CPS in bacterial coaggregation as a receptor polysaccharide, a characteristic seen in other oral streptococci like *S. gordonii* (13, 14), remains a topic of further exploration.

An interesting morphological distinction between the FW213 CPS and pneumococcal capsules is that a deficiency in CPS causes *S. parasanguinis* to form long chains, whereas a defect in the pneumococcal capsule does not result in such a change. The *S. parasanguinis cps* locus possesses a distinct gene, *cpsZ*, which encodes a putative autolysin. CpsZ, similar to its autolysin counterparts, is integral to CPS polymer biosynthesis, cell division, and determination of bacterial chain length. The manifestation of extended bacterial chains is also observed in CPS-deficient lactobacilli (46), hinting at the coupling of cell division and CPS synthesis. The action of CpsZ and other cell wall hydrolases might underpin this linkage, thereby mediating bacterial chain lengths (47). In *S. mutans*, removal of the autolysin gene, *atlA*, results in the formation of elongated chains (48). Just as in the *cpsZ* mutant, biofilm formation is inhibited. In *S. pneumoniae*, mutation in genes responsible for autolysins influences bacterial chain lengths (49). However, an increase in these bacterial chain lengths can promote pneumococcal adhesion (50), suggesting a different fundamental mechanism. For *S. parasanguinis*, the formation of long chains may not be the primary reason for the observed biofilm defect. When CpsZ was overexpressed in CPS mutants, there was a notable decrease in bacterial chain length, but biofilm remained unchanged. It is conceivable that the cell wall-associated CPS plays a direct role in *S. parasanguinis* biofilm formation, functioning independently of the well-characterized biofilm proteins, BapA1 and Fap1. The incorporation of *cpsZ* within the CPS cluster supports the idea that autolysin can potentially modulate cell envelope biogenesis (51). The layout of a CPS locus followed by a *cpsZ*-homologous gene is widely preserved among other oral streptococci (38). It remains to be determined if the *CpsZ* functionality observed in *S. parasanguinis* is mirrored in other oral streptococci. An unanswered question is why *S. parasanguinis* produces CPS but refrains from developing pneumococci-like capsules. The exact encapsulation process in *S. pneumoniae* is yet to be deciphered. CpsZ’s presence might influence the non-encapsulated nature of CPS in *S. parasanguinis*. While CpsZ may help CPS associate with the *S. parasanguinis* cells, autolysin LytA in *S. pneumoniae* makes pneumococcal capsules come off cells in response to antimicrobial peptides (52). Nevertheless, our ongoing studies illuminate a genetic pathway that gives rise to varied CPS configurations and underscores the evolutionary importance of CPS loci in streptococci. This is significant for devising potent vaccines targeting both encapsulated and non-encapsulated pneumococci.

## MATERIALS AND METHODS

### Bacterial strains, culture conditions, and plasmids

Bacterial plasmids and strains used in this study are listed in Table 2. *E. coli* strains, *S. parasanguinis* FW213, *S. pneumoniae*, and their derivatives were cultured as previously described (53). To isolate specific streptococcal mutants, kanamycin was used at a concentration of 125 µg/mL, and erythromycin was used at 10 µg/mL. *E. coli* strains were grown with the appropriate antibiotics, kanamycin at 50 µg/mL and erythromycin at 300 µg/mL.

**TABLE 2:** Plasmids and stains used in this study

Plasmids/strains	Relevant characteristics	Source
Plasmids		
pGEM-T	Cloning TA vector; Amp^r^	Promega
pVPT-TAP	Shuttle vector with maltose promoter; Erm^r^	
∆*cpsE*-pGEM-T	*cpsE* with 342-bp deletion; Amp^r^, Kan^r^	This study
*cpsE*-pVPT	*cpsE* full length cloned in pVPT-TAP; Erm^r^	This study
∆*cpsP*-pGEM-T	*cpsP* with 190-bp deletion; Amp^r^, Kan^r^	This study
*cpsP*-pVPT	*cpsP* full length cloned in pVPT-TAP; Erm^r^	This study
∆*cpsQ*-pGEM-T	*cpsQ* with 216-bp deletion; Amp^r^, Kan^r^	This study
∆*cpsR*-pGEM-T	*cpsR* with 246-bp deletion; Amp^r^, Kan^r^	This study
∆*cpsIQ*-pGEM-T	*cpsIQ* with 1,076-bp deletion; Amp^r^, Kan^r^	This study
∆*cpsRJ*-pGEM-T	*cpsRJ* with 1,318-bp deletion; Amp^r^, Kan^r^	This study
∆*cpsZ*-pGEM-T	*cpsZ* with 616-bp deletion; Amp^r^, Kan^r^	This study
*cpsZ*(1401)-pVPT	*cpsZ* 1–1,401 bp cloned in pVPT-TAP; Erm^r^	This study
*cpsZ*-pVPT	*cpsZ* full length cloned in pVPT-TAP; Erm^r^	This study
*19cS*-pVPT	*19cS* full length from type 19C cloned in pVPT-TAP; Erm^r^	This study
*19fIJ*-pVPT	*19fIJ* full length from type 19F cloned in pVPT-TAP; Erm^r^	This study
*19fI*-pVPT	*19fI* full length from type 19F cloned in pVPT-TAP; Erm^r^	This study
*19fJ*-pVPT	*19fJ* full length from type 19F cloned in pVPT-TAP; Erm^r^	This study
*19aIJ*-pVPT	*19aIJ* full length from type 19A cloned in pVPT-TAP; Erm^r^	This study
*S. parasanguinis* strains		
FW213	Parent strain	
∆*cpsE*	FW213 *cpsE:aphA3*; Kan^r^	This study
∆*cpsE/cpsE*-pVPT	CpsE complement; Kan^r^, Em^r^	This study
∆*cpsP*	FW213 *cpsP:aphA3*; Kan^r^	This study
∆*cpsP/cpsP*-pVPT	CpsP complement; Kan^r^, Em^r^	This study
∆*cpsQ*	FW213 *cpsQ:aphA3*; Kan^r^	This study
∆*cpsR*	FW213 *cpsR:aphA3*; Kan^r^	This study
∆*cpsZ*	FW213 *cpsZ:aphA3*; Kan^r^	This study
∆*cpsZ/cpsZ*(1401)-pVPT	CpsZ (1–1,401 bp) complement; Kan^r^, Em^r^	This study
∆*cpsZ/cpsZ*-pVPT	CpsZ complement; Kan^r^, Em^r^	This study
∆*cpsIQ*	FW213 *cpsIQ:aphA3*; Kan^r^	This study
∆*cpsIQ/19aIJ*-pVPT	Type 19A CpsIJ interchange of FW213 CpsIQ; Kan^r^, Em^r^	This study
∆*cpsIQ/19fIJ*-pVPT	Type 19F CpsIJ interchange of FW213 CpsIQ; Kan^r^, Em^r^	This study
∆*cpsIQ/19fI*-pVPT	Type 19F CpsI interchange of FW213 CpsIQ; Kan^r^, Em^r^	This study
∆*cpsIQ/19fJ*-pVPT	Type 19F CpsJ interchange of FW213 CpsIQ; Kan^r^, Em^r^	This study
∆*cpsRJ*	FW213 *cpsRJ:aphA3*; Kan^r^	This study
∆*cpsRJ/19aIJ*-pVPT	Type 19A CpsIJ interchange of FW213 CpsRJ; Kan^r^, Em^r^	This study
∆*cpsRJ/19fIJ*-pVPT	Type 19F CpsIJ interchange of FW213 CpsRJ; Kan^r^, Em^r^	This study
∆*cpsRJ/19fI*-pVPT	Type 19F CpsI interchange of FW213 CpsRJ; Kan^r^, Em^r^	This study
∆*cpsRJ/19fJ*-pVPT	Type 19F CpsJ interchange of FW213 CpsRJ; Kan^r^, Em^r^	This study
FW213/*19cS*-pVPT/	Type 19C CpsS expressing in FW213; Em^r^	This study
*S. pneumoniae* strains		
19A	Parent strain	This study
19F	Parent strain	This study

### DNA manipulation, construction of mutants, and genetic complementation

Standard methods were used for DNA manipulation. Plasmid DNA was isolated using the Qiaprep Miniprep Kit (Qiagen). PCR was carried out using KOD DNA polymerase (Novagen) with primer pairs listed in Table 3. Nucleotide sequencing was conducted at the DNA sequencing facility of the University of Alabama at Birmingham. Homologous sequence alignment was performed using the Vector NTI software (Invitrogen).

**TABLE 3:** Primers used in this study^*a*^

Primer	Sequence (5’−3’)
*cpsE* 5′	ATGGGAGAAGAAAGAATCG
*cpsE* 3′	CGCTCCTTCTTTCCTAAGC
*cpsE* rev HindIII 5′	GCTCAAGCTTCGAGTATCGTCCTAGTTCCTCTG
*cpsE* rev HindIII 3′	GCTCAAGCTTATAACCGTCACACCAATCAGTTC
*cpsE* comp SalI 5′	GCTCGTCGACATGGGAGAAGAAAGAATCG
*cpsE* comp KpnI 3′	GATCGGTACCCTTCGCTCCTTCTTTCCT
*cpsP* 5′	GGAATTGGATAAAACAACGACC
*cps*P 3′	TTTTCCCAATTGTTGGCATT
*cpsP* rev EcoRI 5′	GCGAATTCATTTTCGCTTATGTTGGGAC
*cpsP* rev EcoRI 3′	GCGAATTCTCGTTGCTGCTCGCTATT
*cpsP* comp SalI 5′	GCTCGTCGACATGAAAAAAATACTTTATGTAA
*cpsP* comp KpnI 3′	GATCGGTACCTCTCAATTCCTCAATTAATT
*cpsQ* 5′	TATTTCATTTTATTTGGACCAGGAA
*cps*Q 3′	ATACGTCCTTAAACCAGCTTCAACA
*cpsQ* rev EcoRI 5′	GCGAATTCCTGAATGGTGTTATAAAGCCACACT
*cpsQ* rev EcoRI 3′	GCGAATTCACAAGAGCAGGGCTGACAAAAG
*cpsR* 5′	TTCTGTGACCCCATCCTCG
*cpsR* 3′	GAAATGCTCGTCTAAAATCGGT
*cpsR* rev EcoRI 5′	GCGAATTCTCGAGAAGATCAACTCTATACC
*cpsR* rev EcoRI 3′	GCGAATTCCAACAATAGAACCTGTTACATG
*cpsZ* 5′	GCGTCATTGCTTGCGGTGAA
*cpsZ* 3′	CCGATACTGGTACGTTTGGCTTA
*cpsZ* rev EcoRI 5′	GATCGAATTCGATTTTATATAGCTGCGGCTCA
*cpsZ* rev EcoRI 3′	GATCGAATTCTCTTTCTCATCGTTCTTCCCTT
*cpsZ* comp SalI 5′	GCTCGTCGACATGTATAAGGGAAGAACGATGAG
*cpsZ* comp SalI 3′	GCTCGTCGACTTGATAACGTGGGCTAGCAAA
*cpsIQ* 5′	ATTTCTGTAGCGCTTTCTGTG
*cpsIQ* 3′	TTTTCCTGAACGGTTGTCTTT
*cpsIQ* rev EcoRI 5′	GCGAATTCAATGGTGTTATAAAGCCACACT
*cpsIQ* rev EcoRI 3′	GCGAATTCAACAACTGATGCCTACAATGAC
*cpsRJ* 5′	TTTATCTCGGTTGAATGTGGC
*cpsR*J 3′	GAAGTCACTTCATCAGCTTTCG
*cpsRJ* rev EcoRI 5′	TCGAATTCGTCATTGCTTGCGGTGAAC
*cpsRJ* rev EcoRI 3′	GCGAATTCTATCTTGGATAGTCCCTTTCC
*19cS* comp SalI 5′	GCTCGTCGACTTGAAGATTGTAATTCCAAGA
*19cS* comp KpnI 3′	GCTCGGTACCTAGATTGTTGTTCATATCTTGC
*19fIJ* comp SalI 5′	GCTCGTCGACATGAGTTATTTATTTTTACT
*19fIJ* comp KpnI 3′	GCTCGGTACCTGATATTTTTTTATGATTTTTAAAGT
*19fI* comp SalI 5′	GCTCGTCGACATGAGTTATTTATTTTTACTTTGCC
*19fI* comp KpnI 3′	GCTCGGTACCTTCTTCTTTTAATTTGATACTTGAG
*19fJ* comp SalI 5′	GCTCGTCGACATGAATACTAAAATTAAAAATATA
*19fJ* comp KpnI 3′	GCTCGGTACCTGATATTTTTTTATGATTTTTAAAG
*19aIJ* comp SalI 5′	GCTCGTCGACATGACTTATTTATTTTTACTC
*19aIJ* comp KpnI 3′	GCTCGGTACCATTTGATGTTTTTTTACTAG

^*a*^ Restriction enzymes are underlined.

To determine the roles of genes in the CPS locus, single or double mutants were created by inserting non-polar kanamycin cassette (*aphA3*) as previously described (54). Mutant strains were confirmed through sequencing analysis of the respective PCR products amplified from corresponding genomic DNAs. Genetic complementation was performed to restore the function of select mutants. Briefly, the shutter vector *tap*-pVPT carrying the full-length ORF of each gene was transformed into the targeted mutants. The transformants were selected based on erythromycin resistance and confirmed through PCR analysis. The validated complemented strains were then evaluated for CPS production and bacterial morphology and biofilm formation as described (54).

### Isolation of polysaccharides

Cell wall polysaccharide from *Streptococcus parasanguinis* FW213 was isolated and purified as the method described previously (55). Briefly, the bacteria were cultured at 37°C until the late stationary phase in a complex medium previously described (56) that contained 0.5% glucose. Cells were harvested from 5-L cultures, rinsed with 10 mM Tris-HCl buffer (pH 8.0) containing 0.1% Triton X-100, and then washed with water. The cells were subsequently suspended in a buffer containing protease and digested with 2.5 mg/mL protease (Sigma) at 50°C for 2 days. After denaturing protease with 6 M Guanidine-HCl, the cells were collected via centrifugation and further digested with 400 U/mL mutanolysin and 2 mg/mL lysozyme (both from Sigma) in 20 mM sodium/potassium phosphate buffer (pH 6.7) that contained 0.5 mM MgCl_2_, 0.5 mM CaCl_2_, and 0.5% azide to cleave any possible peptidoglycans. The proteins were precipitated using 5% (wt/vol) trichloroacetic acid at 4°C and removed by centrifugation. The remaining soluble fractions were neutralized by concentrated Tris. These soluble materials underwent dialyzed against water and then against a 10 mM Tris-HCl buffer (pH 8.0) containing 75 mM NaCl. The solution was then applied to a DEAE Sephacel (GE Healthcare) anion exchange column that had been equilibrated with this buffer. The column was eluted using a linear gradient of NaCl (75–300 mM) in 10 mM Tris buffer. Column fractions were assayed by the phenol sulfuric acid reaction (57). Polysaccharides from the column appeared as a symmetrical peak in fractions ranging from 105 to 145 mM NaCl. These fractions were pooled, dialyzed, and lyophilized for structural NMR studies.

### Structure characterization of CPS by NMR spectroscopy

NMR studies were performed as previously described (58). Before NMR analysis, polysaccharides (3–10 mg) were lyophilized twice from 99.8% D_2_O and then dissolved in 99.996% D_2_O. Spectra were recorded at 25°C using a Bruker DRX-500 spectrometer equipped with a cryoprobe. All proton and carbon chemical shifts were referenced to internal acetone, with δ ^1^H = 2.225 ppm and δ ^13^C = 31.07 ppm.

Multiplicity-edited HSQC was utilized to differentiate methylene from methine groups, aiding in the identification of C6 groups of hexoses and C5 of pentoses. Common homonuclear two-dimensional NMR methods such as double-quantum filtered coherence spectroscopy, total coherence spectroscopy (TOCSY), and nuclear overhauser effect spectroscopy (NOESY) were complemented by the hybrid method HSQC-TOCSY. In the congested carbohydrate spectra, this method was enhanced by high digital resolution in the indirect dimension (^13^C). Another hybrid pulse sequence, HSQC-NOESY, recorded at high digital resolution in ^13^C, proved particularly valuable for correlating C5 of β-Gal*p* with H1 and H4. HMBC spectra were used to identify linkage positions and assignments of residues. All NMR data were processed using NMRpipe and NMRDraw (NMRScience) and analyzed by NMRview (One Moon Scientific).

The ^1^H-^13^C NMR spectrum of the cell wall polysaccharide was assigned using standard techniques of scalar coupling correlation. Starting with the resonances in the anomeric region between 4 and 6 ppm in the ^1^H dimension and 110–90 ppm in the ^13^C dimension, each residue’s resonance was labeled with a capital letter. Other signals were then assigned through scalar correlation. After completing the assignment for each atom, linkage positions were determined using HMBC, correlating both the anomeric ^1^H with the ^13^C of the linkage position and the anomeric ^13^C with the ^1^H of the linkage position.

### Subcellular fraction experiments

Twenty milliliters of bacterial cultures grown to an OD_470_ of 0.8 were harvested by centrifugation at 6, 000 × *g* for 10 minutes. Bacterial cell pellets were separated from culture supernatants. The culture supernatants were precipitated by adding ethanol to a final concentration of 70% and centrifuged. The resulting precipitated pellets were resuspended in 1 mL of TEP buffer as described (59) and used as culture supernatant fraction. The bacterial pellets were resuspended in 1 mL of spheroplasting buffer (10 mM Tris HCl, 2 mM MgCl_2_, 26% raffinose, 1 mM phenylmethylsulfonyl fluoride) along with 100 U of mutanolysin. The supernatant, separated from the spheroplast by centrifugation at 12,000 × *g* for 30 minutes, was used as the cell wall-associated protein fraction. The pellet, resuspended in 1 mL of spheroplasting buffer, was used as the cytoplasmic protein fraction as described previously (60).

### BactELISA and immunoblot analysis

Antisera for *S. pneumoniae* types 4, 12, 19, and 23 were kindly gifted from Dr. Janet Yother. Subtype antisera types 19A, 19F, 19C, and 19B&C were purchased from SSI Diagnostica, Statens Serum Institut, Hilleroed, Denmark. The mouse monoclonal antibody type 19F was produced in Dr. Moon Nahm’s Laboratory. Purified type 19A antibody from human serum was also prepared by Dr. Moon Nahm’s laboratory. Immunosorbent assay (BactELISA) was performed as previously described (53). In brief, streptococcal cells were coated onto 96-well plates and blocked with 1% bovine serum albumin in phosphate-buffered saline (PBS) for 1 hour. The plates were then incubated with primary antibodies for 1 hour and washed three times with PBS containing 0.1% Tween 20. Horseradish peroxidase-conjugated secondary antibodies were incubated for another hour, followed by rinse with PBS containing 0.1% Tween 20. Antibody reactivities were developed using ECL chemiluminescence reagents and quantified at 490 nm using a microplate reader (Biotek).

Immunoblot analysis was performed as previously described (61). Protein or polysaccharide samples were separated by SDS-PAGE and transferred to nitrocellulose membranes. After incubation with primary and secondary antibodies, horseradish peroxidase-conjugated secondary antibodies were detected using ECL chemiluminescence reagent.

### Biofilm assays

*S. parasanguinis* and its variant strains were grown overnight in THB media. The bacteria were diluted at 1:100 ratio into fresh THB containing 1% glucose. Two hundred microliters of diluted cultures were transferred into a saliva-coated 96-well polystyrene microtiter plate (Nunc). Each sample was prepared in triplicate and incubated for 10 hours at 37°C under 5% CO_2_. Biofilms that formed on the plates were stained with 0.1% crystal violet for 15 minutes, and the optical density was measured at 562 nm using a microplate reader (Biotek).

## SUPPLEMENTAL MATERIAL

Supplemental Figures spectrum.01885-23-s0001.pdf Figures S1 to S6.

ASM does not own the copyrights to Supplemental Material that may be linked to, or accessed through, an article. The authors have granted ASM a non-exclusive, world-wide license to publish the Supplemental Material files. Please contact the corresponding author directly for reuse.
