# Supplementary material for: Identification and genetic engineering of pneumococcal capsule-like polysaccharides in commensal oral streptococci
Source: Microbiol Spectr. 2024 Mar 15;12(4):e01885-23. doi: 10.1128/spectrum.01885-23 (PMC10986556; doi:10.1128/spectrum.01885-23)

Supplemental Figure 1. Central part of the ^{1}H-^{13}C HSQC spectrum of the cell wall polysaccharide of *S. parasanguinis* FW213. The capital letters identifying the sugar residues in the spectrum and in Table 1 use the notation as described by Beynon et al., 1992. The chemical shifts for F1 and C2 are aliased by 35.5 ppm in the 13C dimension but are corrected in the Table 1.

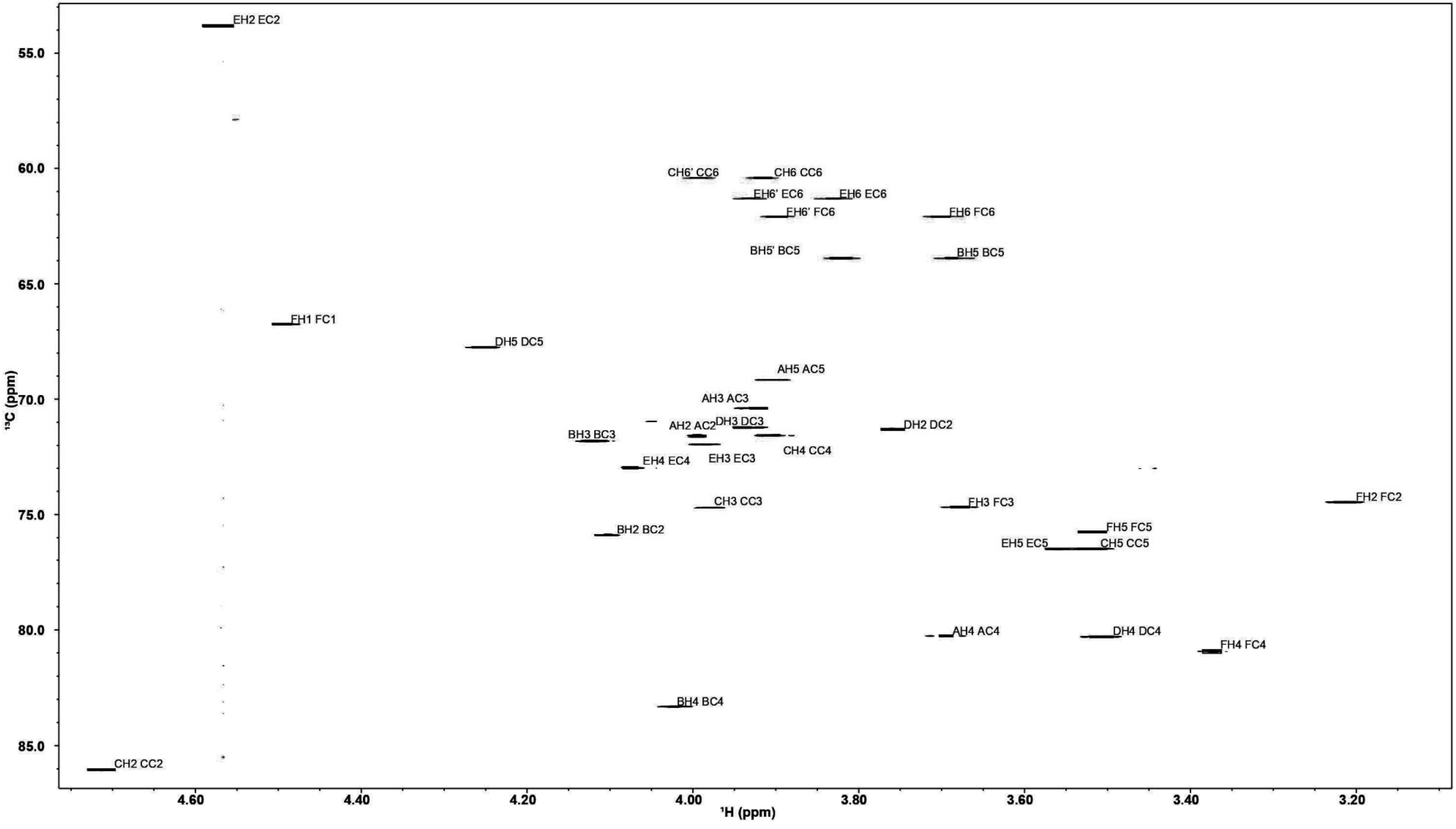

Supplemental Figure. 2

Long chains phenotypes of *cps* derivatives. Derivative strains were grown to OD470nm = 1.0 and observed under phase contrast microscope. Light microscopic views at 40X.

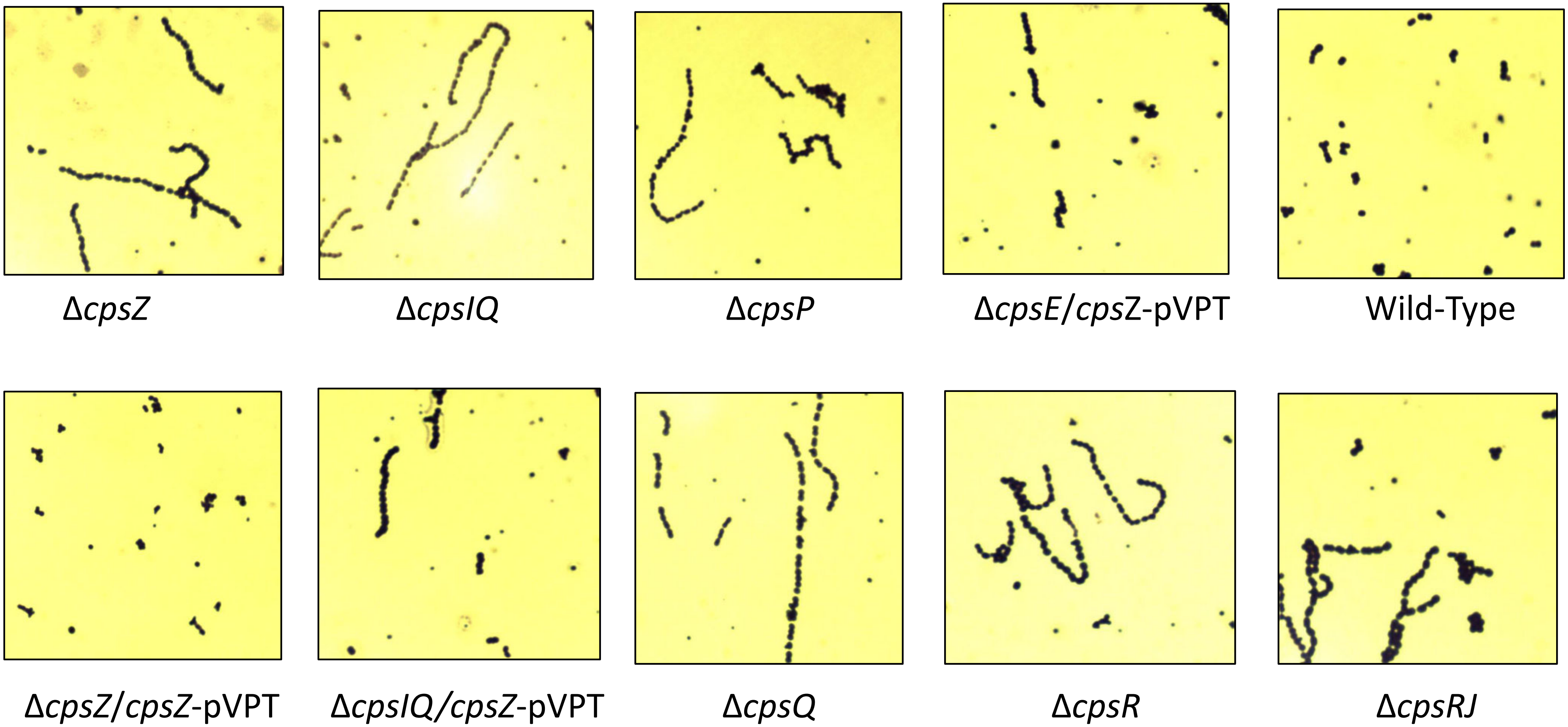

Supplemental Figure. 3.

Structure of FW213 capsular polysaccharides and biosynthetic genes. This is based on predicted functions of CPS gene homologues and current studies. D-Glc, glucose; D-ManNAc, N-acetylmannosamine; L-Rha, rhamnose; D-Rib, ribose.

CpsF ***CpsH*** ***CpsG*** ***CpsF*** ***CpsE***

[→4)-β-D-ManNAc-(1→4)-α-L-Rha(1→PO_4^-→4)-β-D-ManNAc-(1→4)-β-D-Glc-(1→]$_n$

3

↑

1

β-D-Rib-(1→4)-α-L-Rha

CpsP ***CpsQ***

Supplemental Figure. 4
Comparison of CPS loci among *S. parasanguinis* and *S. pneumoniae* 19.

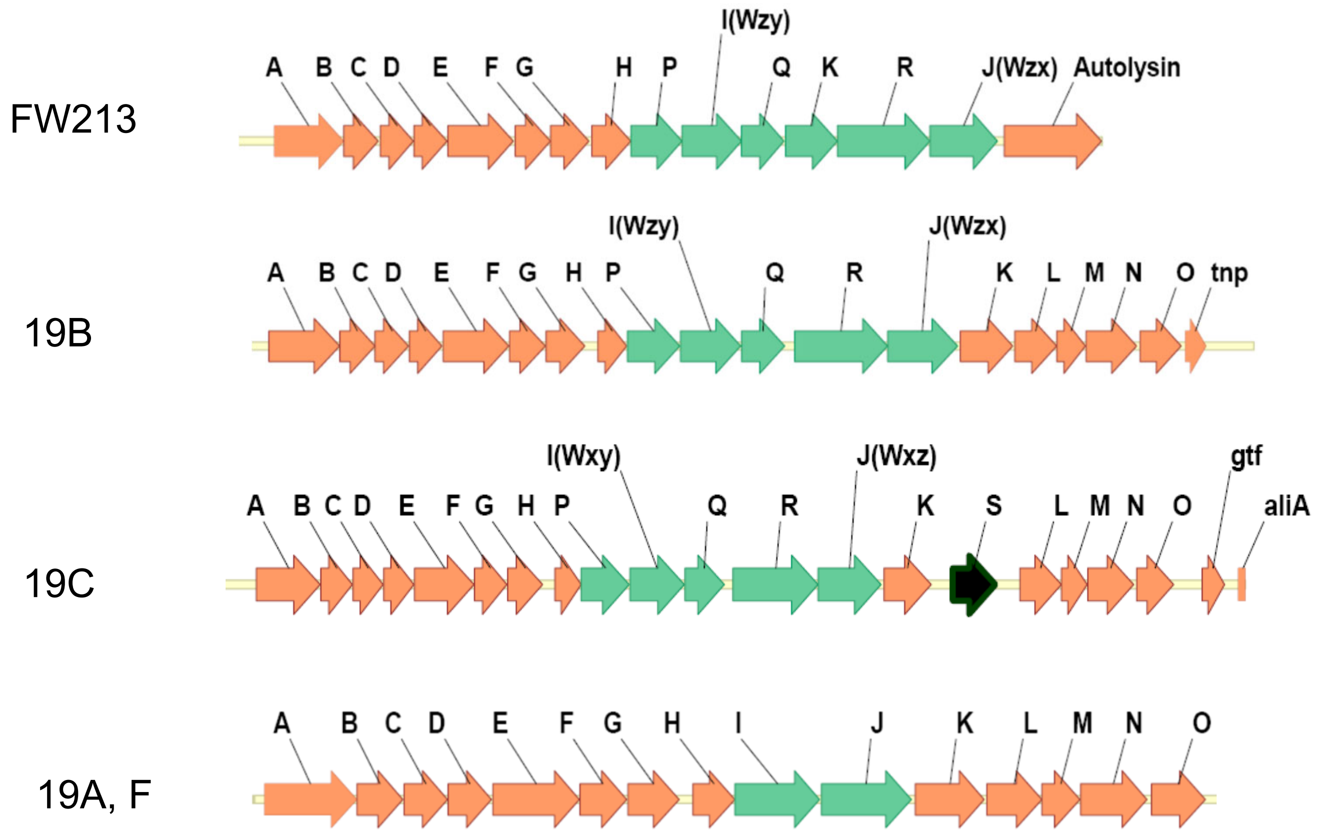

Supplemental Figure. 5

Testing antigenicity of various bacterial strains with antisera type 19.

BactELISA was performed using antisera against *S. pneumoniae* serotype 19 capsules. Samples were analyzed in triplicate and their reactivity was shown in OD values of 490 nm. Error bars represent standard deviations. (*, P ≤ 0.05; **, P ≤ 0.01; ***, P ≤ 0.001; ****, P ≤ 0.0001).

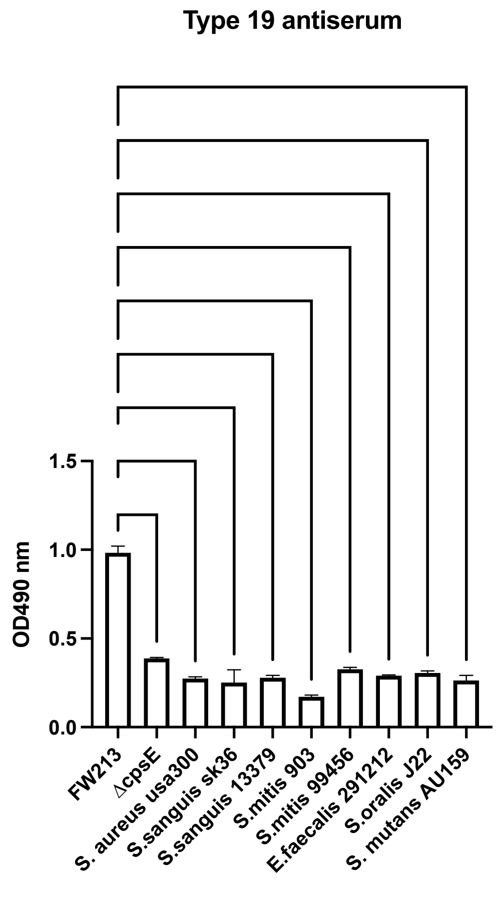

Supplemental Figure. 6
Real-time PCR evaluating *cpsZ* gene expression level in wild type FW213 and ΔCpsE.
SYBR-green qRT-PCR master mix was used to process extracted RNA of bacterial samples. Reference gene 16S rRNA primers and cpsZ-specific primers are designed as following: 16S5 (GAGAGATGGACCTGCGTTGT), 16S3 (GCCGAAGATTCCCTACTGCT), cpsZ5 (GCTGATGAAGTGACTTCAAACGA), and cpsZ3 (GCTACATCTGTTGAGCGACTGG). $\Delta C_T = C_T cpsZ - C_T 16S$, (ns, $P > 0.05$).

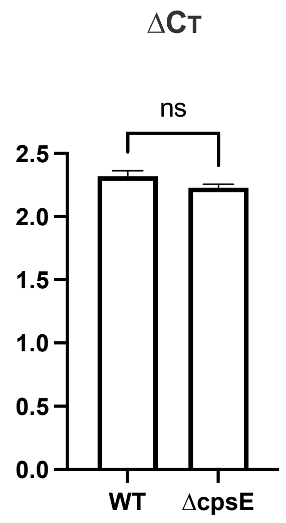

Supplement: Supplemental Figures — Figures S1 to S6. [file spectrum.01885-23-s0001.pdf]
